# A Review of Developments in Polymer Stabilized Liquid Crystals

**DOI:** 10.3390/polym15132962

**Published:** 2023-07-06

**Authors:** Yong Ye, Li Guo, Tingjun Zhong

**Affiliations:** 1 Nanjing M&C Electronic New Material Co., Ltd., Nanjing 211300, China; yeyong0609@163.com; 2 Shanghai Materials Electronics Co., Ltd., Shanghai 201109, China; guolidehaoyou@163.com; 3 Department of Chemistry, College of Science, China Agricultural University, Beijing 100193, China

**Keywords:** polymer stabilization, nematic liquid crystals, cholesteric liquid crystals, blue phase liquid crystals, smectic liquid crystals, ferroelectric liquid crystals, antiferroelectric liquid crystals

## Abstract

Polymer-stabilized liquid crystals (PSLCs) are multi-functional materials consisting of polymer networks in a continuous phase of liquid crystals (LCs), of which polymer networks provide anchoring energy to align the LCs. A number of improvements are detailed, including polymer-stabilized nematic liquid crystals (PSNLCs), polymer-stabilized cholesteric liquid crystals (PSCLCs), polymer-stabilized blue phase liquid crystals (PSBPLCs), polymer-stabilized smectic liquid crystals (PSSLCs), polymer-stabilized ferroelectric liquid crystals (PSFLCs), and polymer-stabilized antiferroelectric liquid crystals (PSAFLCs) in this review. Polymer stabilization has achieved multiple functionalities for LCs; in smart windows, a sufficiently strong electric field allows the LCs to reorient and enables switching from a scattering (transparent) state to a transparent (scattering) state. For broadband reflectors, the reflection bandwidth of LCs is manually tuned by electric fields, light, magnetic fields, or temperature. PSBPLCs open a new way for next-generation displays, spatial light modulators, sensors, lasers, lenses, and photonics applications. Polymer networks in PSFLCs or PSAFLCs enhance their grayscale memories utilized in flexible displays and energy-saving smart cards. At the end, the remaining challenges and research opportunities of PSLCs are discussed.

## 1. Introduction

**Figure 1:**
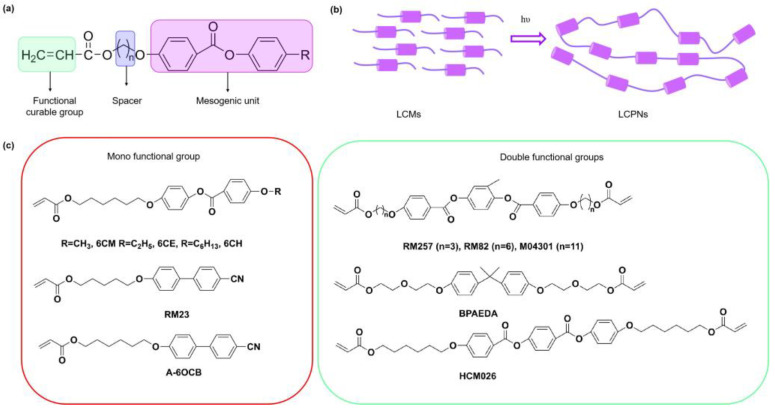
(**a**) Representative structure of LCM examples showing the functional curable group, spacer, and mesogenic unit; (**b**) the preparation mechanism of LCPNs under UV light; (**c**) molecular structures of the LCMs with mono-functional groups and double functional groups.

Liquid crystals (LCs), anisotropic fluids presenting spontaneous orientational order, are famous for their applications in displays, smart windows, photonic crystals, lasers, lenses, diffusers, and augmented reality (AR) technology. The nematic LC (NLC) phase is a common LC state, and shows anisotropic birefringence properties $∆n=n_{e}-n_{o}$ (1), where the $n_{e}$ and $n_{o}$ indicate the extraordinary and ordinary refractive index, respectively. The addition of chiral molecules into NLC phases can result in cholesteric LC (CLC) phases and the occurrence of blue phases (BPs). Polymers are usually utilized as additives in LCs to optimize their electro-optical properties. Polymer-dispersed liquid crystals (PDLCs) and PSLCs are two classical types of polymer/LC composites. PDLCs are mostly fabricated by dispersing LC droplets in a polymer substrate, presenting strong light scattering due to the refractive index difference between the polymers and LCs [1]. PSLCs are composite materials formed by oriented polymer networks and low molecular weight LCs, which are usually fabricated by photopolymerization of a relatively small content (generally <10 wt.%) of photo-curable liquid crystal monomers (LCMs, shown in Figure 1a) dissolved in these low molecular weight LCs. The LCMs are polymerized to liquid crystal polymer networks (LCPNs) by mixing a small number of LCMs into the non-reactive LCs in a homogeneous solution upon UV irradiation. The fabricated films, having excellent thermal, mechanical, dielectric and optical properties, are applied as brightening films in displays to improve energy efficiency (Figure 1b) [2]. The performance and durability of PSLCs are dependent on the structures of formed polymer networks, which are generally affected by structures of LCMs with mono- and double functional groups (Figure 1c), the ratio of LCMs and non-reactive LCs, the curing intensity of UV light, the curing time, and the polymerization temperature. However, the monomers chosen for polymer stabilization are not required to be LCMs [3]. For instance, polystyrene and poly (methyl methacrylate) can stabilize the BP LCs and extend the temperature range of BP stability [4]. The stabilization of the pristine director configuration of LCs is realized in PSLCs as the polymer networks form a template of the LC order [5], or as the macroscopic orientation of LC directors is stabilized by a crosslinked polymer network in PSLCs. The applications of PSLCs are concentrated in the field of smart windows, broadband reflectors, and diffractive optical devices [6].

Generally, without an electric field, the LC molecules in the PSNLCs will be oriented in the direction of a polymer network alignment; with an applied electric field, the LC molecules rotate, the polymer networks impede their rotation, and the optical properties of PSNLCs will be changed, enabling their applications in smart windows, dimming glasses, and microstrip line phase shifters. Dynamic control of the selective reflection of CLCs has been focused on, which is realized by various external stimuli, including thermal, optical, and electrical and magnetic inputs. PSCLCs are fabricated to enhance the response of CLCs to external stimuli, and in the meantime, electrically tunable dynamic optical responses in PSCLCs can be realized by CLCs with negative dielectric anisotropy and the position or bandwidth of the selective reflection changes, showing great potential in the applications of sensors, reflective smart windows, bistable devices, and rewritable papers [7]. Additionally, PSCLCs with photo-isomeric additives can further tune their optical properties and be applied in anti-counterfeiting paintings. If the PSCLCs are photopatterned, an orbital angular momentum (OAM) processor is proposed; when a direct current (DC) electric voltage is applied, the working band of PSCLC is extended and the process is reversible, and the PSCLC OAM-multiplexing holograms are generated and applied as OAM detectors [8]. The three types of BPs, which are BPI, BPII, and BPIII, have attracted considerable interest because they have a fluid lattice, exhibiting electric-field-induced birefringence, a temperature-dependent Kerr constant, and selective reflection of circularly polarized light [9]. As BPs exist in a small temperature range, usually 1–2 °C, the applications of BPs have been limited. To solve this problem, multiple strategies have been executed to widen the thermal phase range of BPs, and an effective way is polymer stabilization. PSBPLCs enable new next-generation BP LC displays (LCDs) with fast response and no surface treatment (e.g., polyimide alignment layer) because of its stabilization for BPs with wide temperature ranges. Additionally, it provides an attractive platform for fast-response transmissive displays, 3-dimensional lasers, and nonlinear optics [10]. PSFLCs are materials that can be applied in optical shutters, privacy protection windows, and photonics [11]. Due to the fast switching times (~ms) of ferroelectric LCs (FLCs) and antiferroelectric LCs (AFLCs), polymer stabilization is utilized for enhancing their mechanical integrity, improving their alignments, and further reducing their response times in displays [12].

In this paper, the research progress on PSLCs is summarized and the innovative applications of PSLCs for improved devices are reviewed. Considering the development of PSLCs, the main challenges in industrial productions, versatile applications, and other potential technologies are prospected.

## 2. PSNLCs

**Figure 2:**
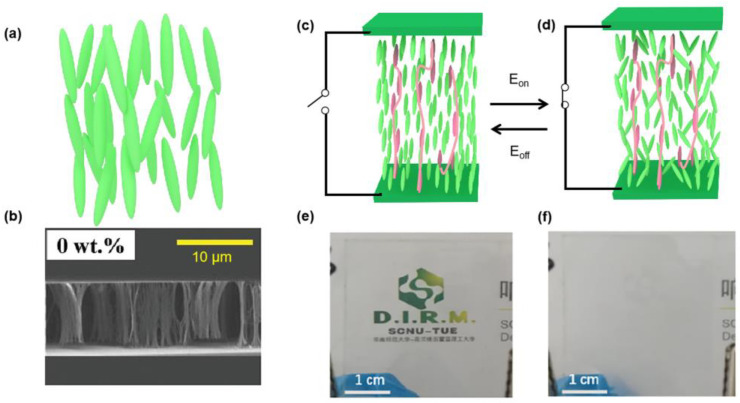
(**a**) Molecular arrangements of NLCs; (**b**) SEM micrography of the polymer networks with 0 wt.% mono-acrylate ratio from cross-section view (Adapted with permission from Y. Zhou, 2020 [14]); device structure of the PSNLC at the off-state (**c**) and the on-state (**d**); the photography of the PSNLC at the off-state (**e**) and the on-state (**f**) (Adapted with permission from X. W. Hu, 2020 [15], the Chinese words underlying the SCNU-TUE are Chinese version of South China Normal University-Eindhoven University of Technology).

NLCs are mostly used in the PSLC fabrication because of their large birefringence and sensitivity to electric, magnetic, optical, and thermal fields (Figure 2a). As PSLCs have a high fluidity, their properties can be tuned by the cross-link density of polymer networks. Moreover, the birefringence of NLCs is preserved after the polymerization of the monomers are doped in LCs; while applying a small electric field, a large photorefractive effect will appear [13]. The SEM micrography of the polymer networks without a mono-functional group LCM is shown in Figure 2b; the formed polymer networks stand vertically between the substrates, presenting a similar tree shape [14]. The working mechanism of the PSNLC device is presented in Figure 2c,d; in the field-off state, by pre-aligning the LC cell, the homeotropic LC state is obtained (Figure 2c). The cell is transparent (Figure 2e) because the refracting index of the NLCs with negative dielectric anisotropy and polymer networks match [15], while in the field-on state, NLCs will form a multi-domain structure, and the refractive index of the LC domains and polymer networks is mismatching. The scattering appears in the PSNLC film (Figure 2f) [15].

PSNLC devices can quickly switch between transparent and scattering states, which are applied as smart windows and optical shutters [14]. Without an electric field, the LCs and polymer network are uniformly aligned and non-scattered. When an electric field is applied across the PSNLC, the LCs reorient and are turned into a poly-domain structure because of the aligning effect of the polymer network. Then, part of the incident light is scattered and comes out of the viewing side of the display [16]. By introducing polymer walls in reverse-mode, PSNLC films with photomasks show excellent stability regarding their electro-optical properties [17]. As PSNLC is always limited in visible light, antimony doped tin oxide (ATO) nanoparticles (NPs) are added to shield more than 80% of the infrared radiation [18]. For NLCs, the presence of vapors lowers the clearing temperature of LCs, and under polarized optical microscopy (POM), the optical behavior of LCs switches from bright to dark. Based on PSNLCs, a method for obtaining a 2-dimenisonal (2D) profile of toluene vapor in a microchannel is developed by controlling the concentration of toluene vapor in a microchannel. The interference colors of the PSNLC under POM are tuned, then the diffusion coefficient of dissolved toluene inside the PSNLC is measured to be 1.01 × 10^−6^ cm^2^·s^−1^ [19]. The constituent molecules of NLCs have permanent dipole moments; as a DC voltage is applied, the reflection symmetry along the LC director is broken, and a net polarization is produced [20]. The introduction of polymer networks in the PSNLCs leads to higher driving voltage, while the particle-laden PSLC has decreased the threshold voltage and response times. A PSNLC device incorporating functionalized single-walled carbon nanotubes (SWCNTs) has been developed as the CNT addition reduces the threshold voltage [21]. A novel polarizing light waveguide plate (LWGP) from PSNLCs is produced for converting unpolarized light into linearly polarized light [22].

## 3. PSCLCs

**Figure 3:**
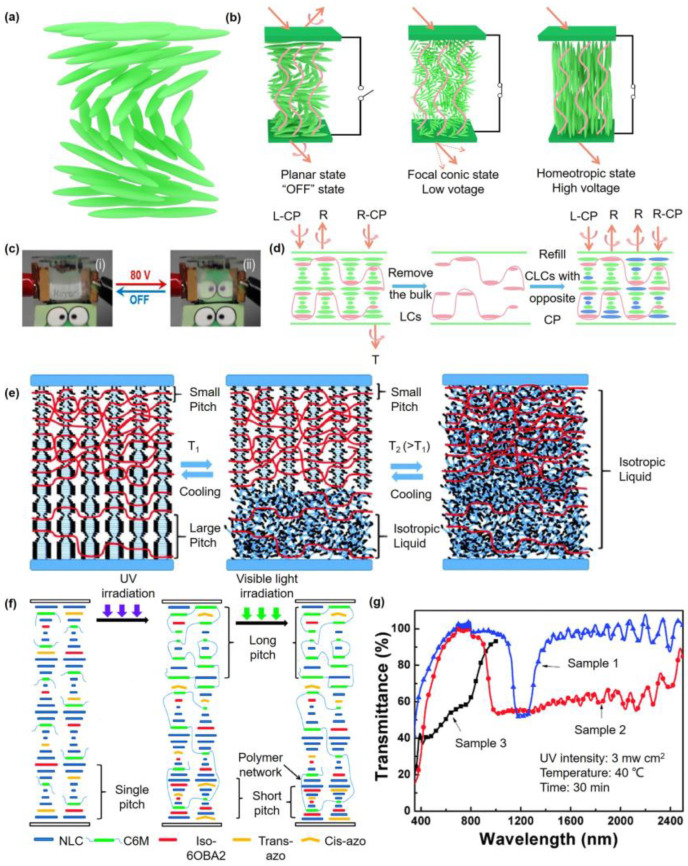
(**a**) Helical structure of CLC phase; (**b**) schematic diagram of the operation of the PSCLC light shutter; (**c**) photographs demonstrating the reflection/transmission of the LH/RH PSCLC assembly without an electric field and with the application of an electric field at 80 V (Adapted with permission from K. M. Lee, 2014 [29]); (**d**) schematic preparation method for the PSCLC film, R: reflection, T: transmission; (**e**) schematic diagram presenting the mechanism for a thermal switchable bandwidth broadband reflector (Adapted with permission from H. Khandelwal, 2016 [32]); (**f**) the preparation process and mechanism of the broadband reflection of a PSCLC (Adapted with permission from X. W. Chen, 2014 [34]); (**g**) the transmittance spectra of the PSCLCs after UV irradiation (Adapted with permission from X. W. Chen, 2014 [34]).

CLCs in the planar orientation assemble into a helical structure, which exhibits selective reflection because of the 1-dimensional (1D) bandgap (shown in Figure 3a). Bragg reflection of circularly polarized light with the reflection band gap is given by: ∆λ=ΔnP (2), where P is the helical pitch that is defined by the concentration, $P={\left(HTP[C]\right)}^{-1}$ (3), and the HTP is the helical twisting power of the chiral dopant [23]. External stimuli including temperature, electric fields, pressure, magnetic fields, and light can tune the pitch of CLCs, changing the position of the selective reflection. Additionally, with the application of an electric field, the helical pitch of CLCs is also adjusted by utilizing interdigitated electrodes. As a result, CLCs play a critical role in optical sensors, as the changes of optical information can be observed with our naked eyes [24].

Similar to CLCs with positive dielectric anisotropy, as the LCM or non-LCM is polymerized in the planar state, the fabricated polymer networks are parallel to the substrates, and the planar state is stabilized. The application of an electric field can turn the PSCLC from the planar state (reflective) to the focal conic state (scattering) or the homeotropic state (clear) (seen in Figure 3b). Due to the elastic restoring force of polymer networks, PSCLC turns into its pristine planar state without an electric field and avoids the formation of a metastable focal conic state [25]. If the homeotropic state is stabilized at first, the formed polymer networks are perpendicular to the substrates; when increasing the temperature, the homeotropic state changes into the focal conic state; by the application of an electric field, the homeotropic state returns [26]. Light shutters from PSCLCs are always utilized for greenhouse windows. Bistable devices based on PSCLCs have been extensively explored due to their energy-saving advantages; with an electrochromic layer, four operating states exist—transparent, light-scattering, colored transparent, and colored light-scattering—which can be regulated by the alternating frequency and the electric field direction [27].

Recently, electrical tuning of the pitch can be realized in PSCLCs. Large and stable electrically regulated control of the bandwidth of the spectral reflection of PSCLCs has been mostly reported, and the reflection notch of CLCs has been broadened as much as 300%. Because of the electromechanical displacement mechanism originating from trapped ions, switching times of the PSCLCs are slower than mechanisms based on LC reorientation [25]. For PSCLCs with negative dielectric anisotropy, upon the application of an electric field, LCs cannot reorient and keep the reflective state; blue-shifting tuning of the selective reflection of PSCLCs is shown, which is fully reversible [28]. Color-switchable mirrors originating from electrically tunable bandwidth broadening of the circularly polarized (CP) reflection of PSCLCs are fabricated (Figure 3c), dynamically turning the selective reflection (colored) to a broadband reflection (mirror). Before applying the DC electric field, the word “mirror” is presented; with the application of a DC bias of 80 V, the word is invisible and the mirror-like reflection is observed [29]. By utilizing a wash-out/refill method (described in Figure 3d), PSCLCs with a double-handed CP reflection band are prepared, and both right-circularly polarized (R-CP) and left-circularly polarized (L-CP) light are reflected [30]. Firstly, the mixture consisting of monomers and LCs in the cell is under UV irradiation, then the cell is washed out using cyclohexane and tretrahydrofuran to remove the non-polymerized LCs. After drying the cell, another kind of LC is refilled into the cell, then the PSCLC film structure is built by both the permanent superstructure of the polymer network and the CLCs with opposite helical structure. Then, both R-CP and L-CP are reflected [30]. The LC director rotates around a preferred direction (helical axis) in CLCs, while in the oblique helicoid (ChOH) state, the director forms a tilt angle <90° with the helical axis. The pitch and tilt angle of the LC director relative to the helical axis is dependent on the electric field strength [31].

The PSCLC film can used to prepare a broadband reflector. The polymer network density is higher at the top side (short pitch side) because of the polymerization-induced diffusion. Through the application of lower voltage or temperature T1 (depicted in Figure 3e), the CLCs with a lower polymer network density will homeotropically reorient, while CLCs confined in the high polymer density remain unchanged. Then, by decreasing the reflection in the longer wavelength without changing the shorter wavelength reflection, further enhancing the electric field or temperature (T2), most CLCs will homeotropically reorient. The longer wavelength reflection will disappear, and the narrowband reflector will form [32]. In conclusion, the reflection bandwidth is manually controlled by both electric fields and temperature [32]. Considering the thermotropic nature of CLCs, the reflection of PSCLC is thermally switched when the CLCs are heated from the LC state to the isotropic liquid state. Based on the CLC showing a twist inversion at a critical temperature Tc, the fabricated PSCLC film reflects a dual CP band in the infrared range when the environment temperature is below Tc. Then, by quenching the film below melting point, the reflection properties can be kept for a long time at room temperature (RT) [33].

As azobenzene compounds such as the chiral bis(azo) molecule (QL76) or azobenzene right-handed chiral dopant 2C can adjust the orientation of LCs, the reflection wavelength of a chiral azobenzene compound doped PSCLC can be extended to cover the 1000–2400 nm range [34,35]. The possible mechanism of the broadband reflection of PSCLC film is depicted in Figure 3f; at the beginning, the composites are filled into the LC cell, and after UV irradiation, the cell is placed upon visible light. The transmittance spectra of three samples (ratios of 2C in samples 1, 2, and 3 are 0 wt.%, 0.6 wt.%, and 0.6 wt.%, respectively, while the ratios of chiral dopant in samples 1, 2, and 3 are 2.55 wt.%, 2.13 wt.%, and 5.70 wt.%, respectively) are shown in Figure 3g. The reflection wavelength of the 2C-doped PSCLC film is broadened, which is in the range of 1000–2400 nm in sample 2, while that of sample 1 without 2C is in the range of 1130–1310 nm. With the increasing chiral dopant, the reflection wavelength of sample 3 can be tuned to the visible light region covering 400–800 nm. Compared to electro-tuning, the polymer stabilization of azo-CLCs can also decrease the time during both the photoinduced isotropic state and photo-displaced CLC phase [36]. By doping fluorescent molecules into PSCLCs and constructing a gradient pitch, distinctive circularly polarized luminescence (CPL) emissions from two surfaces of one LC cell is realized. Additionally, the reflection bandwidth of PSCLCs from 150 to 500 nm is broadened, and thus the CPL signals are changed [37]. Except for broadband reflection, paraboloid-shape microlens arrays consisting of PSCLCs exhibit their special polarization-dependent optical properties, which have advantages in sensing polarization states of input lights [38]. Dispersing CLC droplets in the polymer solution, oblate CLC droplets are formed upon water evaporation by 3-dimensional (3D) printing, and various bioinspired stimuli-responsive systems are achieved due to the intriguing properties of oblate CLC droplets such as color change and shape deformation [39]. In addition, CLC droplets are dispersed in polymer films using innovative methods. As the films are mechanically stretched, spherical CLC droplets can turn into an oblate shape, then the selective Bragg reflection region increases and the structural color presents itself, which is applied in smart sensors [40]. With superior mechanical properties of polymer substrates, CLC droplets show great potential for applications in smart functional materials, photonic devices, sensors, and soft robotics [41].

## 4. PSBPLCs

**Figure 4:**
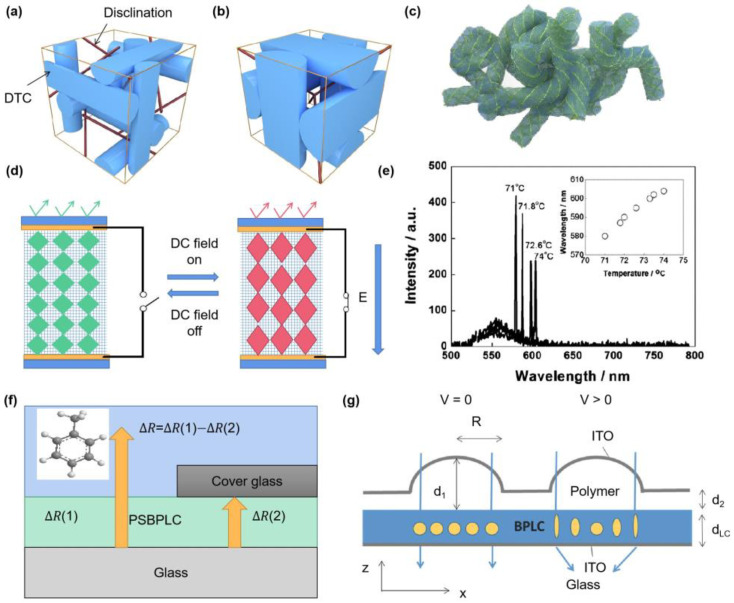
Schematic illustrations of the structures of (**a**) BPI, (**b**) BPII, and (**c**) BPIII (Adapted with permission from S. S. Gandhi, 2015 [45]); (**d**) schematics of the possible mechanism for the DC electric field-switchable band-gap shifting in PSBPLC; (**e**) Typical laser emission spectra at enhanced temperatures for the PSBPLC film, with the peak lasing wavelengths at various temperatures shown inside (Adapted with permission from K. Kim, 2015 [49]); (**f**) schematic illustration of PSBPLC films to vapor; (**g**) mechanism for the microlens from PSBPLCs with a curved electrode (Adapted with permission from Y. Li, 2016 [53]).

BPLCs are high chirality LCs with self-assembled 3D-structure-presenting cubic-crystalline symmetries, which attract much attention and apply in various fields containing photonic crystals, displays, and mirrorless lasers because of their photonic bandgaps (PBGs) for visible light originating from self-assembled crystalline structures [42]. BPLCs occur in a narrow temperature close to their clearing points, and they have a special three-dimensional (3D) structure consisting of double twist cylinders and disclinations, which are divided into three types: BPI, BPII, and BPIII (Figure 4a–c) [43]. BPI is a body-centered cubic of double twist cylinder (DTC) assembly, BPII is simple cubic, and BPIII is amorphous with an entangled double-twisted LC cylinder arrangement and has isotropic symmetry [44,45]. Generally, each BP is stable only in a narrow temperature range, while PSBPLC opens a new pathway to extend the stability of BPs. In 2002, H. Kikuchi et al. demonstrated the stabilization of BPLCs using polymer networks over a temperature range of more than 60 K, including room temperature [46]. Polymer-stabilized BPII presents a wide temperature range of 50 °C, including room temperature [47]. PSBPLCs are focused on for their applications in optical devices, and next-generation LCDs based on PSBPLCs show fast electro-optical response and need no surface treatment [48]. However, LCDs based on PSBPLCs need a lower driving voltage for their worldwide applications. BPLCs are sensitive to external fields containing temperature, light, and electric fields. When applying a DC electric field across the PSBPLC film, a striking phenomenon happens; a weak electric field of 1.33 V/μm makes the photonic bandgap move from 560 nm to 680 nm, turning the green film to a red-shifted one due to the field-induced lattice distortion (depicted in Figure 4d). When increasing the electric field to 2.67 V/μm, the band gap can be switched to about 220 nm from the original wavelength. The high-tunable PSBPLCs will open a new method for applied photonics, but aside from that, PSBPLCs are regarded as an optimal choice for lasers on the basis of uniform BPII with mono-platelet domains. Typical laser emission spectra at enhanced temperatures of PSBPLC film are exhibited in Figure 4e. The wavelengths of peaks lasing at various temperatures are visible in the small graph; as the temperature increases from 71.2 to 75 °C, the laser peak shifts towards longer wavelengths due to the large shift for PBG [49]. Additionally, the PSBPLC laser exhibits stable emission with the temperature range extending 15 °C containing RT [42]. A dynamically tunable diffraction grating from monodomain PSBPLCs is demonstrated, and the diffraction efficiency is improved by 9% [50].

PSBPLCs generate an optical response to volatile organic compounds (VOCs) such as toluene (Figure 4f), which is more sensitive than the PSNLCs or PSCLCs [51]. Putting PSBPLCs in a vacuum chamber and introducing vapor to a pressure, the PSBPLC film reaches an equilibrium state for some minutes while the other half of the polymerized film is masked by cover glass, preventing vapor exposure. Finally, the optical responses (reflectance R intensities) of films obtained from regions 1 and 2 at exposure times 0 and t are recorded and expressed as: ∆R=∆R(1)−∆R(2) (4), exhibiting an optical response to toluene vapor in a broad concentration range (140~5155 ppm) [51]. Because of the unique structural nature of BPLCs, such as stimuli-responsive Bragg reflection-bandgap and fast electro-optical Kerr switching, PSBPLCs have been widely utilized for multiple applications such as lasers, different optoelectronic devices, and tunable photonic devices and lenses [52]. A microlens from PSBPLC proposed by employing a curved ITO electrode is shown in Figure 4g [53]. With a polymer layer below the curved electrode, a non-uniform electric field is produced and polarization independence is obtained; by shaping the curved electrode, the phase of the lens is optimized for obtaining a parabolic shape. When the electric field is off, the refractive index of PSBPLC presents as uniform and isotropic, leading to zero optical power [53,54]. Therefore, phase profile is flat and the incident light will go through the LC cell by keeping its original direction; by applying an electric field between the two ITO electrodes, a non-uniform vertical field is produced in the PSBPLC layer. As the cell gap is the largest in the center of lens, the electric field strength is the weakest, and the produced birefringence is the smallest. In contrast, if the largest induced birefringence is at the field with the thinnest cell gap, then the refractive index is reduced and a positive lens phase is formed [53,54].

## 5. PSSLCs

**Figure 5:**
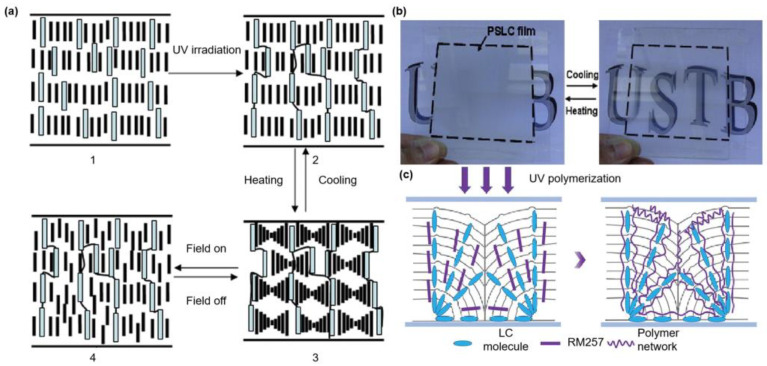
(**a**) Schematic illustrations of PSSLC film preparation (Adapted with permission from G. H. Pan, 2008 [56]). (**b**) Photographs of PSSLC film as the environmental temperature is over and below the phase transition point of SmA to CLC (Adapted with permission from G. H. Pan, 2009 [57]); (**c**) Schematic presentation of the UV polymerization process of polymer-stabilized TFCD arrays (Adapted with permission from J. B. Wu, 2022 [59]).

The most studied mesophases in smectic LCs (SLCs) are the smectic A (SmA) and smectic C (SmC) phases. For the SmA phase, the director orientation is parallel to the normal layer K, while the director orientation is tilted to the normal layer by an angle in the SmC phase [55]. Like CLCs, the planar molecular alignment in the SmA phase is also stabilized with polymer networks. G. H. Pan et al. have fabricated PSLC films switching between a transparent state at lower-temperature environment and a light-scattering state at a higher-temperature environment based on the LC host with an SmA-CLC phase transition [56,57,58]. Firstly, the mixture of photo-curable LC monomer, non-curable LC monomer and chiral dopant is filled into the LC cell (Figure 5a, step 1). By cooling the mixture to the chiral SmA phase, the LC molecules are homeotropically oriented. With UV irradiation (Figure 5a, step 2), the homeotropically oriented polymer networks form, and the film is transparent. By increasing the environmental temperature, the film changes to a light-scattering state, causing the chiral SmA phase to transfer to the focal conic state of the CLC phase (Figure 5a, step 3). Upon the application of a sufficient electric field, the strongly light-scattering state changes into a transparent one as the LC molecules are oriented by the electric field (Figure 5a, step 4). Photographs of PSSLC film are shown when the environmental temperature is below and above the SmA phase to CLC phase transition point; the film is transparent and scattered, respectively (Figure 5b) [57]. Consequently, the PSSLC films can be applied in light shutters, E-papers, alarm devices for overheating protection, and thermoelectrical sensors [56,57,58]. Recently, in the SmA phase, the direct distribution of focal conic domains (TFCDs) is generated with the polymer stabilization strategy. With UV irradiation, a polymer network is produced (Figure 5c), enabling the polymer-stabilized smectic hierarchical architectures to be applied as microlens arrays [59].

## 6. PSFLCs and PSAFLCs

**Figure 6:**
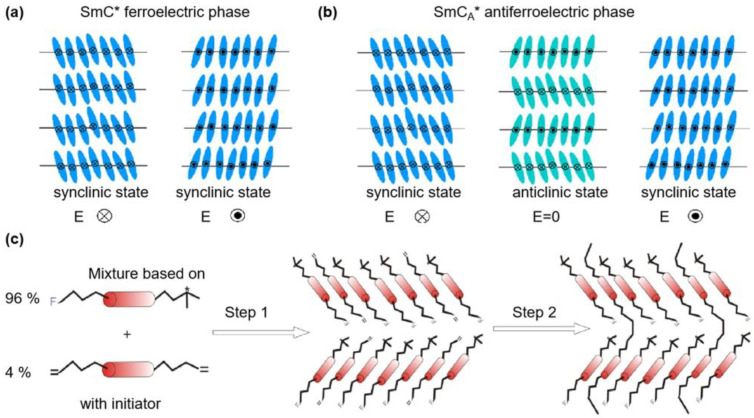
Schematic illustrations of the molecular structures of (**a**) ferroelectric phase (at zero field, two stable states) and (**b**) antiferroelectric phase (at zero field, one stable state; with an electric field, two synclinal states) (adapted with permission from E. Dmochowska, 2021 [60]); (**c**) Schematic illustrations of the fabrication process of the PSAFLC with a hypothetic molecular arrangement where * means the chiral part (adapted with permission from M. Czerwiński, 2019 [61]).

FLCs and AFLCs are chiral SmC (SmC*) LCs whose molecules are tilted at angle θ to the layer normal z (Figure 6a). Spontaneous electric polarizations happen in FLCs, and the polarization direction can be reversed by an electric field [60,61,62]. For AFLCs (Figure 6b) without an electric field, LC molecules present an anti-clinical orientation in adjacent layers. With the application of a sufficient electric field, one of two ferroelectric states is displayed [60,61,62]. The fabrication process for polymer stabilization of AFLC materials is shown in Figure 6c: mixing the AFLC and curable monomer in an isotropic phase (step 1), putting the solution into LC cell, then upon UV irradiation, monomers are polymerized into polymer networks at room temperature (step 2) [61]. Polymer networks have a significant effect on the FLC, as 10–15% of free energy changes. When the polymer concentration is only 3 wt.%, the SmC* phase stability is not affected. Compared to the original Bragg wavelengths, the Bragg wavelengths of PSFLCs will decrease because of polymer network shrinkage [12,63]. The effect of polymer networks on the dielectric properties of FLCs has been studied extensively. Two major collective dielectric modes of FLCs are Goldstone and soft modes; J. Hemine et al. have found that the increasing polymer content leads to decreased strength of Goldstone-mode relaxation [64,65]. Utilizing polymer networks in electrically suppressed helix FLC (abbreviated as ESHFLC), Y. Ma et al. have fabricated an LC cell whose response time is less than 30 μs and the contrast ratio is more than 10,000:1 [66].

## 7. Conclusions

Polymer stabilization has attracted much attention in LC research since it was demonstrated in 1992. The polymer stabilization technique has great potential in the applications of tunable lasers, optical sensors, and LCDs. Electrically, thermally, and optically switchable PSLC devices are designed and fabricated to optimize performance, which demonstrates the importance of polymers for advanced LC technologies. For the challenge of smart windows fabricated by PSLCs, multi-modulation and environment-adaptation are needed to obtain comfortable indoor conditions and satisfy different requirements of customers. For example, programmable and colorful-patterned PSLC films are required for practical applications such as room decoration. By introducing new LCMs, stronger anchoring energy between polymer networks and LC molecules is achieved without enhancing the operating voltage of PSLC films, which is advantageous for stable and scalable industrial production. Polymer stabilization has always increased the threshold voltage of LCs. By incorporating it with other systems, including particle-laden and polymer dispersion, a better electro-optical performance of PSLC can be realized. By introducing various fluorescent molecules into PSLCs, CPL performance is combined with selective Bragg reflection of the PSCLC films. By designing structures of PSCLCs, CPL materials with large dissymmetry factors and high emission quantum yields are achieved, which have potential for applications in information storage and processing. For PSCLC lasers, they have advantages of a wide tunable range, a short response time, and long-term stability, which all have potential applications in optoelectronic devices. For PSBPLC lasers, a broad-temperature laser can be obtained through the addition of dyes or other compounds. Generally, dynamic tunable photonic bandgaps in PSBPLCs still need to be improved due to their unwanted reflections. With good polarization selectivity and wavelength selectivity, PSBPLCs can play a vital role in augmented reality (AR) displays. Electro-mechanical behaviors of polymer networks make PSLCs applicable in sensors and actuators. However, making PSLCs simple and large-scale industrial fabrication is still a major problem because of the weak bonding between LCs and substrates. Moreover, the film’s durability and environmental stability need to be further improved for the needs of energy saving and environmental protection. In the meantime, driving voltages of PSLCs are high and response time is slow.

## 8. Outlook

Except for smart windows, broadband reflectors, and other photonic and optic applications, by incorporating 2-dimenisonal (2D) nanostructured LCs into polymer networks, water-treatment membranes can be fabricated to improve water permeation based on their high rejection of ions, organic molecules, and other pollutants. Moreover, LCs are ionic conductors; when combining the mechanical properties of polymers, PSLCs have outstanding potential for sensors, actuators, and polymer electrolytes. In addition, with the controlled molecular arrangements of LCPNs, tissue growth can be controlled because cells are alike active nematics, which have advantages over muscle tissue engineering.

## Data Availability

Not applicable.

## References

[1] Mucha M. (2003). Polymer as an important component of blends and composites with liquid crystals. Prog. Polym. Sci..

[2] Lee K.M., Ware T.H., Tondiglia V.P., McBride M.K., Zhang X.P., Bowman C.N., White T.J. (2016). Initiatorless photopolymerization of liquid crystal monomers. ACS Appl. Mater. Interfaces.

[3] Radka B.P., Lee K.M., Godman N.P., White T.J. (2022). Electro-optic characteristics of stabilized cholesteric liquid crystals with non-liquid crystalline polymer networks. Soft Matter.

[4] Kasch N., Dierking I., Turner M. (2013). Stabilization of the liquid crystalline blue phase by the addition of short-chain polystyrene. Soft Matter.

[5] Dierking I. (2004). Fractal and non-fractal structure-property relationships of polymer-stabilized liquid crystals. Adv. Funct. Mater..

[6] Li X.S., Guo Y.Q., Zhang M.S., Zhang C., Niu R., Ma H.M., Sun Y.B. (2023). Colorable light-scattering device based on polymer-stabilized ion-doped cholesteric liquid crystal and an electrochromatic layer. ACS Appl. Mater. Interfaces.

[7] Lee K.M., Tondiglia V.P., White T.J. (2016). Photosensitivity of reflection notch tuning and broadening in polymer stabilized cholesteric liquid crystals. Soft Matter.

[8] Xu C.T., Zhang D.W., Yuan R., Chen Q.M., Liang X., Hu W. (2023). Optical orbital angular momentum processors with electrically tailored working bands. Laser Photonics Rev..

[9] Wang L., He W.L., Wang Q., Yu M.N., Xiao X., Zhang Y., Ellahi M., Zhao D.Y., Yang H., Guo L. (2013). Polymer-stabilized nanoparticle-enriched blue phase liquid crystals. J. Mater. Chem. C.

[10] Chen C.W., Li C.C., Jau H.C., Yu L.C., Hong C.L., Guo D.Y., Wang C.T., Lin T.H. (2015). Electric field-driven shifting and expansion of photonic band gaps in 3D liquid photonic crystals. ACS Photonics.

[11] Lahiri T., Majumder T.P. (2012). The effect of cross-linked chains of polymer network on the memory states of polymer stabilized ferroelectric molecules. Polymer.

[12] Singh U., Spink M., Davis F., Mitchell G. (2015). Morphology of polymer networks formed in the chiral and non-chiral phases of an antiferroelectric liquid crystal. Chem. Phys. Lett..

[13] Wiederrecht G.P., Wasielewski M.R. (1998). Photorefractivity in polymer-stabilized nematic liquid crystals. J. Am. Chem. Soc..

[14] Zhou Y., You Y.X., Xiao X.L., Liu W., Zhou L., Zhang B.B., Zhao W., Hu X.W., Zhang L.Y., Yang H. (2020). Effect of polymer network topology on the electro-optical performance of polymer stabilized liquid crystal (PSLC) devices. Macromol. Chem. Phys..

[15] Hu X.W., Zhang X.M., Yang W.M., Jiang X.F., Jiang X.S., Haan L.T., Yuan D., Zhao W., Zheng N., Jin M.L. (2020). Stable and scalable smart window based on polymer stabilized liquid crystals. J. Appl. Polym. Sci..

[16] Shin Y.H., Jiang J.H., Qin G.K., Wang Q., Zhou Z.Y., Yang D.K. (2020). Patterned waveguide liquid crystal displays. RSC Adv..

[17] Li H., Xu J.J., Ren Y.X., Han R., Song H., Huang R., Wang X., Zhang L.Y., Cao H., Zou C. (2023). Preparation of highly durable reverse-mode polymer-stabilized liquid crystal films with polymer walls. ACS Appl. Mater. Interfaces.

[18] Zhang Z.B., Zhang R.C., Xu L.G., Li J.J., Yang L., Yang Y.N., Bolshakov A., Zhu J.Q. (2021). Visible and infrared optical modulation of PSLC smart films doped with ATO nanoparticles. Dalton Trans..

[19] Liu Z.D., Luo D., Yang K.L. (2020). Monitoring the two-dimensional concentration profile of toluene vapors by using polymer-stabilized nematic liquid crystal in microchannels. Lab Chip.

[20] Hicks S.E., Hurley S.P., Yang Y.C., Yang D.K. (2013). Electric polarization frozen by a polymer network in nematic liquid crystals. Soft Matter.

[21] Prasad S.K., Baral M., Murali A., Jaisankar S.N. (2017). Carbon nanotube reinforced polymer-stabilized liquid crystal device: Lowered and thermally invariant threshold with accelerated dynamics. ACS Appl. Mater. Interfaces.

[22] Moheghi A., Nemati H., Yang D.K. (2015). Polarizing light waveguide plate from polymer stabilized liquid crystals. Opt. Mater. Express.

[23] Nemati H., Liu S.Y., Zola R.S., Tondiglia V.P., Lee K.M., White T., Bunning T., Yang D.K. (2015). Mechanism of electrically induced photonic band gap broadening in polymer stabilized cholesteric liquid crystals with negative dielectric anisotropies. Soft Matter.

[24] Mulder D.J., Schenning A.P.H.J., Bastiaansen C.W.M. (2014). Chiral-nematic liquid crystals as one dimensional photonic materials in optical sensors. J. Mater. Chem. C.

[25] Radka B.P., Pande G.K., White T.J. (2023). The contribution of network elasticity to electro-optic response in polymer stabilized cholesteric liquid crystals. Soft Matter.

[26] Bao R., Liu C.M., Yang D.K. (2009). Smart bistable polymer stabilized cholesteric texture light shutter. Appl. Phys. Express.

[27] Gruzdenko A., Dierking I. (2023). Electro-optic properties of polystyrene particle-laden polymer-stabilized liquid crystals. J. Mater. Chem. C.

[28] Lee K.M., Tondiglia V.P., Godman N.P., Middleton C.M., White T.J. (2017). Blue-shifting tuning of the selective reflection of polymer stabilized cholesteric liquid crystals. Soft Matter.

[29] Lee K.M., Tondiglia V.P., McConney M.E., Natarajan L.V., Bunning T.J., White T.J. (2014). Color-tunable mirrors based on electrically regulated bandwidth broadening in polymer-stabilized cholesteric liquid crystals. ACS Photonics.

[30] Guo J.B., Yang H., Li R., Ji N., Dong X.M., Wu H., Wei J. (2009). Effect of network concentration on the performance of polymer-stabilized cholesteric liquid crystals with a double-handed circularly polarized light reflection band. J. Phys. Chem. C.

[31] Rumi M., Bunning T.J., White T.J. (2018). Polymer stabilization of cholesteric liquid crystals in the oblique helicoidal state. Soft Matter.

[32] Khandelwal H., Timmermans G.H., Debije M.G., Schenning A.P.H.J. (2016). Dual electrically and thermally responsive broadband reflectors based on polymer network stabilized chiral nematic liquid crystals: The role of crosslink density. Chem. Commun..

[33] Agez G., Mitov M. (2011). Cholesteric liquid crystalline materials with a dual circularly polarized light reflection band fixed at room temperature. J. Phys. Chem. B.

[34] Chen X.W., Wang L., Chen Y.J., Li C.Y., Hou G.Y., Liu X., Zhang X.G., He W.L., Yang H. (2014). Broadband reflection of polymer-stabilized chiral nematic liquid crystals induced by a chiral azobenzene compound. Chem. Commun..

[35] White T.J., Bricker R.L., Natarajan L.V., Tabiryan N.V., Green L., Li Q., Bunning T.J. (2009). Phototunable azobenzene cholesteric liquid crystals with 2000 nm range. Adv. Funct. Mater..

[36] White T.J., Bricker R.L., Natarajan L.V., Serak S.V., Tabiryan N.V., Bunning T.J. (2009). Polymer stabilization of phototunable cholesteric liquid crystals. Soft Matter.

[37] Li Z.Z., Lan R.C., Bao J.Y., Hu W., Wang M., Zhang L.Y., Yang H. (2022). Tunable circularly polarized luminescence with a high dissymmetry factor emitted from luminogen-bonded and electrically controlled polymer-stabilized cholesteric liquid crystals. ACS Appl. Mater. Interfaces.

[38] Perera K., Padmini H.N., Mann E., Jάkli A. (2021). Polymer stabilized paraboloid liquid crystal microlenses with integrated pancharatnam-berry phase. Adv. Opt. Mater..

[39] Yang C.J., Wu B.H., Ruan J., Zhao P., Chen L., Chen D., Ye F.F. (2021). 3D-printed biomimetic systems with synergetic color and shape responses based on oblate cholesteric liquid crystal droplets. Adv. Mater..

[40] Yang C.J., Wu B.H., Ruan J., Zhao P., Shan J.Z., Zhang R., Yoon D.K., Chen D., Liu K. (2022). Mechanochromic responses of cholesteric liquid crystal droplets with nanoscale periodic helical structures showing reversible and tunable structural color. ACS Appl. Polym. Mater..

[41] Yang C.J., Chen D. (2022). Researches and applications of cholesteric liquid crystal droplets. Chin. J. Liq. Cryst. Disp..

[42] Choi H.J., Bae J.H., Bae S.W., Lee J.J., Nishikawa H., Araoka F., Choi S.W. (2019). Development of a liquid crystal laser using a simple cubic liquid crystalline blue phase platform. RSC Adv..

[43] Higashiguchi K., Yasui K., Kikuchi H. (2008). Direct observation of polymer-stabilized blue phase I structure with confocal laser scanning microscope. J. Am. Chem. Soc..

[44] Lan Y.F., Tsai C.Y., Lu J.K., Sugiura N. (2013). Mechanism of hysteresis in polymer-network stabilized blue phase liquid crystal. Polymer.

[45] Gandhi S.S., Li Y., Luo D., Chien L.C. (2018). Laser emission in a 3D nanoporous polymer replica of amorphous blue phase III. J Polym. Sci. Pol. Phys..

[46] Kikuchi H., Yokota M., Hisakado Y., Yang H., Kajiyama T. (2002). Polymer-stabilized liquid crystal blue phases. Nat. Mater..

[47] Jo S.Y., Jeon S.W., Kim B.C., Bae J.H., Araoka F., Choi S.W. (2017). Polymer stabilization of liquid-crystal blue phase II toward photonic crystals. ACS Appl. Mater. Interfaces.

[48] Iwata T., Suzuki K., Amaya N., Higuchi H., Masunaga H., Sasaki S., Kikuchi H. (2009). Control of cross-linking polymerization kinetics and polymer aggregated structure in polymer-stabilized liquid crystalline blue phases. Macromolecules.

[49] Kim K., Hur S.T., Kim S., Jo S.Y., Lee B.R., Song M.H., Choi S.W. (2015). A well-aligned simple cubic blue phase for a liquid crystal laser. J. Mater. Chem. C.

[50] Manda R., Pagidi S., Heo Y.J., Lim Y.J., Kim M.S., Lee S.H. (2020). Polymer-stabilized monodomain blue phase diffraction grating. Adv. Mater. Interfaces.

[51] Yang Y., Kim Y.K., Wang X., Tsuei M., Abbott N.L. (2020). Structural and optical response of polymer-stabilized blue phase liquid crystal films to volatile organic compounds. ACS Appl. Mater. Interfaces.

[52] Wu P.C., Chen H.L., Rudakova N.V., Timofeev I.V., Zyryanov V.Y., Lee W. (2018). Electro-optical and dielectric properties of polymer-stabilized blue phase liquid crystal impregnated with a fluorine-containing compound. J. Mol. Liq..

[53] Li Y., Huang S.J., Zhou P.C., Liu S.X., Lu J.G., Li X., Su Y.K. (2016). Polymer-stabilized blue phase liquid crystals for photonic applications. Adv. Mater. Technol..

[54] Li Y., Wu S.T. (2011). Polarization independent adaptive microlens with a blue-phase liquid crystal. Opt. Express.

[55] Dierking I., Mitov M., Osipov M.A. (2015). Smectic layer instabilities in liquid crystals. Soft Matter.

[56] Pan G.H., Yu L.L., Zhang H.B., Guo J.B., Guo R.W., Cao H., Yang Z., Yang H., Zhu S.Q. (2008). Effects on thermo-optical properties of the composition of a polymer-stabilised liquid crystal with a smectic A-chiral nematic phase transition. Liq. Cryst..

[57] Pan G.H., Cao H., Guo R.W., Li W.B., Guo J.B., Yang Z., Huang W., He W.L., Liang X.K., Zhang D.W. (2009). A polymer stabilized liquid crystal film with switching characteristics between light transmission and adjustable light scattering. Opt. Mater..

[58] Pan G.H., Cao Y.B., Guo R.W., Cheng H.C., Yang Z., Guo J.B., Liang X.K., Zhang D.W., Cao H., Yang H. (2009). Effects of the preparing condition of a polymer-stabilised liquid crystal with a smectic-A-chiral nematic phase transition on its properties. Liq. Cryst..

[59] Wu J.B., Wu S.B., Cao H.M., Chen Q.M., Lu Y.Q., Hu W. (2022). Electrically tunable microlens array enabled by polymer-stabilized smectic hierarchical architectures. Adv. Opt. Mater..

[60] Dmochowska E., Herman J., Czerwiński M., Stulov S., Bubnov A., Kula P. (2021). Self-assembling behaviour of chiral calamitic monoacrylates targeted for polymer stabilization of polar smectic phases in chiral liquid crystals. J. Mol. Liq..

[61] Czerwiński M., Urbańska M., Bennis N., Rudquist P. (2019). Influence of the type of phase sequence and polymer-stabilization on the physicochemical and electro-optical properties of novel high-tilt antiferroelectric liquid crystalline materials. J. Mol. Liq..

[62] Dierking I. (2000). Polymer network-stabilized liquid crystals. Adv. Mater..

[63] Dierking I. (2010). Recent developments in polymer stabilised liquid crystals. Polym. Chem..

[64] Hemine J., Kaaouachi A.E., Ismaili M., Douali R., Legrand C., Daoudi A. (2021). Electro-optic and dielectric properties of polymer networks stabilised short pitch chiral smectic C* liquid crystal. Liq. Cryst..

[65] Dierking I. (2014). A review of polymer-stabilized ferroelectric liquid crystals. Materials.

[66] Ma Y., Srivastava A.K., Guo Q., Chigrinov V.G., Kwok H.S. (2014). 29.4: Polymer Stabilized Electrically Suppressed Helix Ferroelectric Liquid Crystal. Dig. Tech. Papers.

